# Electronic and Optical Properties of Atomic-Scale Heterostructure Based on MXene and MN (M = Al, Ga): A DFT Investigation

**DOI:** 10.3390/nano11092236

**Published:** 2021-08-30

**Authors:** Kai Ren, Ruxin Zheng, Peng Xu, Dong Cheng, Wenyi Huo, Jin Yu, Zhuoran Zhang, Qingyun Sun

**Affiliations:** 1 School of Mechanical and Electronic Engineering, Nanjing Forestry University, Nanjing 210037, China; Ruxin.Zheng@institutionalemail.cn (R.Z.); Peng.Xu@institutionalemail.cn (P.X.); dongtian176@163.com (D.C.); WenyiHuo@institutionalemail.cn (W.H.); 2 School of Materials Science and Engineering, Southeast University, Nanjing 211189, China; JinYu@institutionalemail.cn; 3 Center for More-Electric-Aircraft Power System, Nanjing University of Aeronautics and Astronautics, Nanjing 211106, China; Zhuoran.Zhang@institutionalemail.cn

**Keywords:** two-dimensional materials, Hf_2_CO_2_, heterostructure, first-principles calculation

## Abstract

After the discovery of graphene, a lot of research has been conducted on two-dimensional (2D) materials. In order to increase the performance of 2D materials and expand their applications, two different layered materials are usually combined by van der Waals (vdW) interactions to form a heterostructure. In this work, based on first-principles calculation, some charming properties of the heterostructure constructed by Hf_2_CO_2_, AlN and GaN are addressed. The results show that Hf_2_CO_2_/AlN and Hf_2_CO_2_/GaN vdW heterostructures can keep their original band structure shape and have strong thermal stability at 300 K. In addition, the Hf_2_CO_2_/MN heterostructure has I-type band alignment structure, which can be used as a promising light-emitting device material. The charge transfer between the Hf_2_CO_2_ and AlN (or GaN) monolayers is 0.1513 (or 0.0414) |*e*|. The potential of Hf_2_CO_2_/AlN and Hf_2_CO_2_/GaN vdW heterostructures decreases by 6.445 eV and 3.752 eV, respectively, across the interface. Furthermore, both Hf_2_CO_2_/AlN and Hf_2_CO_2_/GaN heterostructures have remarkable optical absorption capacity, which further shows the application prospect of the Hf_2_CO_2_/MN heterostructure. The study of this work provides theoretical guidance for the design of heterostructures for use as photocatalytic and photovoltaic devices.

## 1. Introduction

Since 2004, Novoselov and Geim prepared graphene from graphite by the mechanical exfoliation method [1], and its remarkable physical and chemical properties were explored [2,3,4,5,6,7,8,9,10,11], which also attracted extensive interest and attention on other two-dimensional (2D) materials, and they all show fantastic properties [12,13,14,15,16,17]. For example, black phosphorene is a honeycomb-like folded layered material that can achieve transistor performance with a thickness of less than 7.5 nm, and the highest carrier mobility can be obtained by 1000 cm^2^/V·s when the thickness is 10 nm at room temperature [18,19,20,21]. Puckered arsenene possesses the ability to adjust its bandgap by applying the external strain on its surface. Interestingly, arsenene can even be transformed into a straight gap semiconductor by applying 1% strain [22,23,24,25,26,27]. Transition metal dichalcogenides (TMDs) materials are layered materials with excellent thermal [28,29], electronic [30] and optical properties [31]. For instance, MoS_2_ has high broadband gain (up to 13.3), detection rate (up to 10^10^ cm Hz^1/2^/W) and high thermal stability when using it as an optoelectronic device [32]. In addition, there are Janus TMDs materials that destroy the symmetry of the original structure and make its carrier mobility increase from 28 to 606 cm^2^/V·s [33]. The Janus MoSSe material is able to separate light-generated electrons and holes while also exhibiting perfect light absorption capabilities, which lays the foundation for promoting water redox reactions, and it is a remarkable water decomposition light catalyst [34]. The novel properties of these 2D materials can provide unprecedented help for the development of nano-devices and solar cells [35,36].

In order to expand the application of 2D materials and build more special performances, superposing different layered materials to construct a heterostructure is usually realized [37,38,39,40,41,42,43]. Especially, two-layer materials construct a heterostructure by van der Waals (vdW) forces, which can induce novel interfacial [44], optical [45] and electronic properties [46]. The SiC/TMDs vdW can be used as a water decomposition catalyst to completely separate hydrogen and oxygen under the condition of light [47]. The PbSe/CdSe heterostructure has near infrared emission characteristics, which is closely related to its type-I alignment band [48]. The average carrier value of the type-I PbI_2_/WS_2_ layered heterostructure is 0.039 cm^2^·s^−1^, and it is found that the interlayer diffusion behavior between electrons and holes is similar [49]. These investigations have demonstrated that type-I heterostructures possess promising applications in photocatalytic, photovoltaic and optical devices [50,51]. Recently, layered MAX phases have been exfoliated into monolayer and multilayers, named MXenes, which has attracted wide attention [52]. The charming electrochemical [53], conductive [54] and stable capacity [55] characteristics provide potential applications in electrocatalysts, photocatalysts and energy storage devices [56,57,58]. Although most of the MXenes are metallic, some MXenes are semiconductors with a desirable bandgap [59,60]. In particular, the Cr_2_TiC_2_ monolayer behaves as a novel bipolar antiferromagnetic semiconductor showing opposite spin directions, which can be used as antiferromagnetic spin field effect transistor [61]. Hf_2_CO_2_ possesses excellent electronic and thermoelectric properties and carrier mobility (about 1531.48 cm^2^/V·s for electrons), suggesting an efficient photocatalyst for water splitting and nano-electronic devices [62,63,64,65,66], and this novel electronic characteristic can even be tuned by external strain [67]. Hf_2_CO_2_ is also sensitive to NH_3_, which can sharply enhance electronic conductivity [68]. More recently, MN (M = Al, Ga) has been reported to have remarkable optical, electronic and mechanical properties, and it can be considered as a candidate for future optical and photovoltaic devices [69,70,71,72,73,74,75]. Interestingly, the prepared 2D AlN shows great promise in deep-ultraviolet optoelectronic applications, ultraviolet LEDs and laser diodes [76,77]. In addition, 2D GaN is also fabricated by epitaxial graphene using a migration-enhanced encapsulated growth method [75], and is studied as a decent semiconductor for heterostructures [78], photocatalysts [79] and photocathodes [80]. Moreover, some Hf_2_CO_2_ and MN-based heterostructures have been reported, such as Hf_2_CO_2_/WS_2_ [62], Hf_2_CO_2_/blue phosphorene [65], MoS_2_/MN [81], GeC/GaN [82], etc., while studies of the Hf_2_CO_2_/MN heterostructure are still limited. Therefore, considering such fantastic electronic properties of Hf_2_CO_2_, and the synthesized MN (M = Al, Ga), it is worth constructing the heterostructure by Hf_2_CO_2_ and MN to explore the novel performances and the potential applications.

In this study, the first-principles method was utilized to investigate the formed heterostructure based on Hf_2_CO_2_ and MN (M = Al, Ga). The binding energy and the ab initio molecular dynamics (AIMD) calculations were conducted to check the stability of the heterostructure. Furthermore, the type-I band alignment of the Hf_2_CO_2_/MN heterostructure was addressed, which demonstrates the potential applications of the light-emitting devices. In addition, the charge difference between the MXene and the MX layers was studied and the potential drop was also calculated to develop the interfacial properties of the heterostructure. Moreover, the light absorption capacity of the Hf_2_CO_2_/MN heterostructure was obtained by calculating the intrinsic optical absorption spectrum.

## 2. Computing Method

The method of calculations in this work was based on density functional theory (DFT), implemented by first-principles simulation under the circumstances of the Vienna *ab initio* simulation package (VASP) [83]. Based on generalized gradient approximation (GGA), the Perdew–Burke–Ernzerhof (PBE) functional was employed for the explanation of the exchange correlation functional [84,85,86]. For the more precise bandgap results, we used the hybrid Heyd–Scuseria–Ernzerhof (HSE06; screening parameter 0.2 Å^−1^, mixing parameter 0.25) simulations [87] and the DFT-D3 method of Grimme, and the dipole corrections were also used to correct the weak dispersion forces. A tested 500 eV cutoff energy was considered. After the convergence test for the *k*-point (seen in Table S1 of Supplementary Materials), the Monkhorst–Pack *k*-point of 7 × 7 × 1 was adopted to relax the structure, while the static and optical calculations were conducted by the 11 × 11 × 1 *k*-point. For the prevention of the interaction of the adjacent atomic layers, the vacuum slab was controlled by 25 Å. Furthermore, the energy of the calculated materials in this work was set within 1 × 10^−^^5^ eV, while the Hellmann–Feynman forces on the atoms were set to less than 0.01 eV·Å^−^^1^.

## 3. Results and Discussions

**Figure 1:**
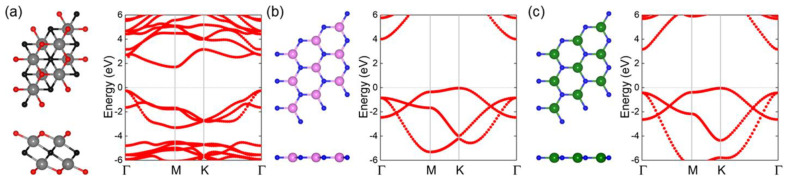
The atomic structure and the band structure of the (**a**) Hf_2_CO_2_, (**b**) AlN and (**c**) GaN monolayers. The grey, red, black, pink, green and blue marks are Hf, O, C, Al, Ga and N atoms, respectively, and the Fermi energy level is 0, shown by gray dashes.

We first optimized the structures of Hf_2_CO_2_, AlN and GaN, and the top and side views of the crystal structure and band energy for the Hf_2_CO_2_, AlN and GaN monolayers are shown in Figure 1a–c, respectively. The lattice constants of monolayered Hf_2_CO_2_ AlN and GaN are obtained by 3.363, 3.127 and 3.255 Å, respectively. In addition, the bond lengths of Hf−C, Hf−O, Al−N and Ga−N in Hf_2_CO_2_, AlN and GaN monolayers are 2.369, 2.132, 1.805 and 1.895 Å, respectively. In addition, the HSE06 method-calculated energy band structures of Hf_2_CO_2_, AlN and GaN monolayers show that all these layered materials have semiconductor features with the bandgaps of 1.820, 4.042 and 3.203 eV, respectively. For the Hf_2_CO_2_ monolayer, the conduction band minimum (CBM) is located at the M point, while the valence band maximum (VBM) appears at the Γ point. The CBM and VBM of AlN (or GaN) are generated at the Γ point and K point, respectively. All these calculated results are almost the same as the previous investigation results [65,66,88].

**Figure 2:**
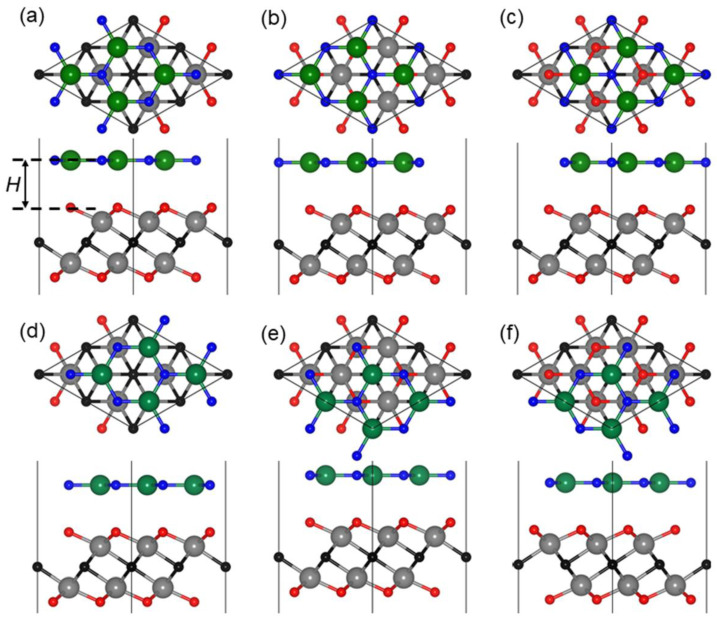
Top and side views of the (**a**) AA, (**b**) AB, (**c**) AC, (**d**) AD, (**e**) AE and (**f**) AF stacking configurations of the Hf_2_CO_2_/MN heterostructure.

To construct the heterostructure by Hf_2_CO_2_ and MN (N = Al, Ga) monolayers, the six most representative stacking configurations, shown in Figure 2, should be taken into consideration. In the six Hf_2_CO_2_/MN heterostructures, the binding energy (*E*_binding_) of the Hf_2_CO_2_/MN heterostructure is decided by:

(1) $$E_{\mathrm{binding}}=E_{\mathrm{MXene}/\mathrm{MN}}-E_{\mathrm{MXene}}-E_{\mathrm{MN}}$$

where *E*_MXene/MN_, *E*_MXene_ and *E*_MN_ show the total energy of the Hf_2_CO_2_/MN heterostructure, monolayered Hf_2_CO_2_ and MN, respectively. The smaller the binding energy, the more stable the structure of the heterostructure [89], and thus the most stable structure of those six stacking configurations of the heterostructure is decided as the lowest binding energy, which is demonstrated in the AA stacking style of the Hf_2_CO_2_/MN heterostructure. The calculated *E*_binding_ of the Hf_2_CO_2_/AlN and Hf_2_CO_2_/GaN heterostructures with the most stable configuration is −56.98 and −52.44 meV/Å^−^^2^, respectively, in Table 1. It is worth noting that the framework of the quantum theory of atoms in molecules (QTAIM) functional is not considered here, which is also a popular method for simulations [90,91,92,93,94,95,96,97,98,99]. The results show that the Hf_2_CO_2_/MN heterostructure is formed by vdW interactions [100,101]. For the most stable Hf_2_CO_2_/AlN and Hf_2_CO_2_/GaN vdW heterostructures, the bond lengths of Hf−C, Hf−O and M−N are slightly changed compared with original layered materials, which further proves the weak vdW forces between the interface of the heterostructures. In addition, the interface distances of the Hf_2_CO_2_/AlN and Hf_2_CO_2_/GaN vdW heterostructures are 1.924 and 2.235 Å, respectively. Additionally, in the following sections, we only discuss the most stable Hf_2_CO_2_/MN heterostructure stacking structure.

**Figure 3:**
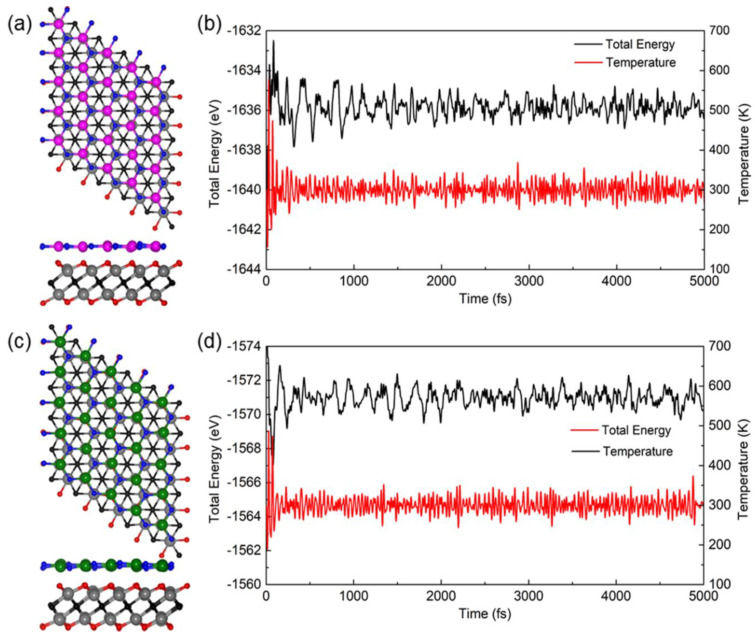
The calculated AIMD snapshots of the (**a**) Hf_2_CO_2_/AlN and (**c**) Hf_2_CO_2_/GaN vdW heterostructures at 300 K by 5 ps, and the monitoring of the energy and the temperature during the AIMD simulation for the (**b**) Hf_2_CO_2_/AlN and (**d**) Hf_2_CO_2_/GaN vdW heterostructures, respectively.

In order to further investigate the thermal stability of the Hf_2_CO_2_/MN vdW heterostructure, AIMD simulations were explored for the Hf_2_CO_2_/MN vdW heterostructure by the Nosé–Hoover heat bath scheme [102]. To consider the constraints of the lattice translation, we constructed a 6 × 6 × 1 supercell for the Hf_2_CO_2_/AlN and Hf_2_CO_2_/GaN vdW heterostructures in the AIMD simulation, which contained 252 atoms in total. The ambient temperature of the simulation was set as 300 K, and the structures of the Hf_2_CO_2_/AlN and Hf_2_CO_2_/GaN vdW heterostructures after relaxation for 5 ps are shown in Figure 3a,c, respectively. The simulation results of AIMD show that the structures of the Hf_2_CO_2_/AlN and Hf_2_CO_2_/GaN vdW heterostructures still remain intact after 5 ps under 300 K, revealing the robust thermal stability of the heterostructure. In addition, as shown in Figure 3b,d, the total energy fluctuation and simulation time for Hf_2_CO_2_/AlN and Hf_2_CO_2_/GaN vdW heterostructures in AIMD calculation are demonstrated, respectively, and they all show the convergence state by time, ensuring the reliability of the results.

**Figure 4:**
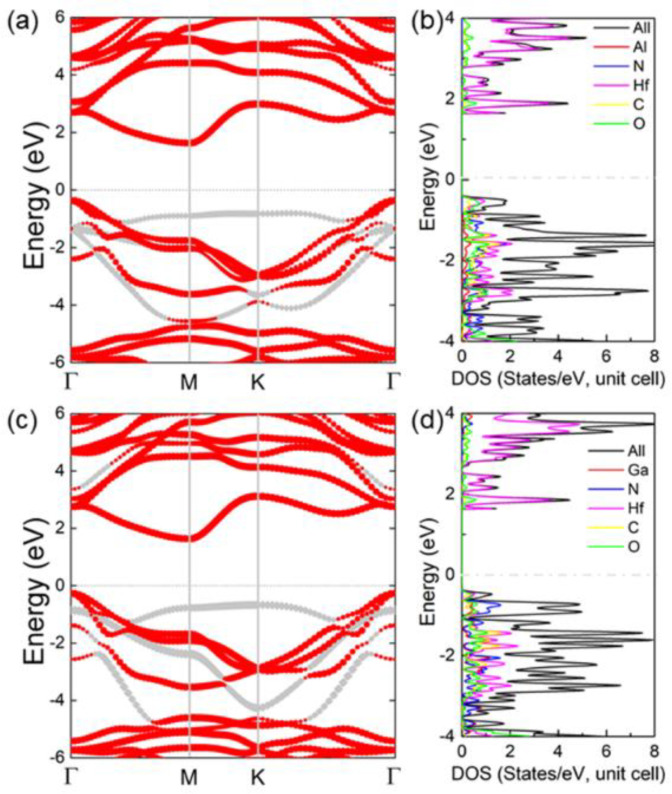
The calculated projected band structure of the (**a**) Hf_2_CO_2_/AlN and (**c**) Hf_2_CO_2_/GaN vdW heterostructures; the projected density of states of the (**b**) Hf_2_CO_2_/AlN and (**d**) Hf_2_CO_2_/GaN vdW heterostructures. The energy of the Fermi is 0.

Figure 4a,c show the projected band structure of the MXene/MN vdW heterostructure. It is obvious that Hf_2_CO_2_/AlN and Hf_2_CO_2_/GaN heterostructures have an indirect bandgap of 2.006 eV and 1.899 eV, respectively. The gray and red marks represent the band contribution of AlN (or GaN) and Hf_2_CO_2_ layers, respectively. Therefore, we can see that the CBM and VBM of both MXene/MN vdW heterostructures are donated from the Hf_2_CO_2_ layer, and the MXene/MN vdW heterostructure shows I-type band structure. In addition, we also investigated the partial density calculation of MXene/MN vdW heterostructures, as shown in Figure 4b,d, which further proves the intrinsic type-I band structure characteristic. The band structures of the MXene/AlN and MXene/GaN vdW heterostructures by all six different stacking configurations are calculated in Figures S1 and S2, respectively, in the Supplementary Materials.

**Figure 5:**
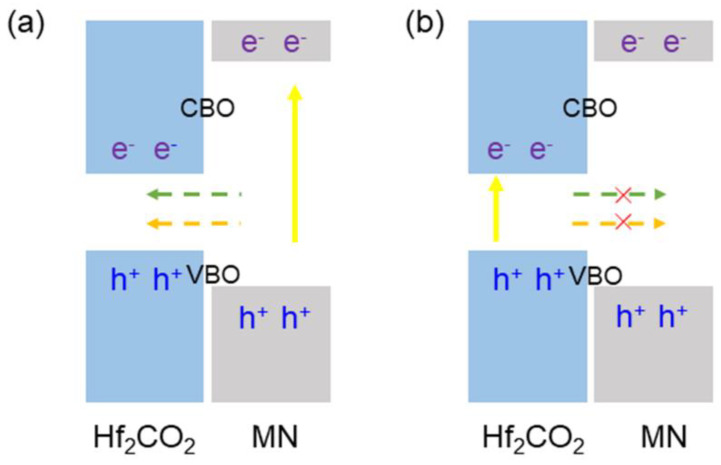
Schematic of the carrier transport in the interface of the type-I band structure for the Hf_2_CO_2_/MN vdW heterostructure. (**a**) Feasible and (**b**) restricted charge transfer path.

In the Hf_2_CO_2_/MN vdW heterostructure, the bandgap of the Hf_2_CO_2_ layer is smaller than that of the AlN (or GaN) layer. Additionally, the CBM and VBM of the MXene/MN vdW heterostructure are fixed in the band gap of the Hf_2_CO_2_ layer, as shown in Figure 5. When some external conditions are applied, the electrons in the wide bandgap of MN will be excited and move to the CBM of MN. At the same time, holes will be induced in the VBM of MN. With the help of conduction band shift (CBO) and valence band shift (VBO), the electrons and holes are excited from the MN layer to the Hf_2_CO_2_ layer at CBM and VBM, respectively, as shown in Figure 5a. The CBO and VBO of the Hf_2_CO_2_/AlN (or Hf_2_CO_2_/GaN) vdW heterostructure are obtained as 2.496 eV (or 0.432eV) and 1.744 eV (or 0.383 eV), respectively. Due to the lower energy, the electrons and holes excited in the Hf_2_CO_2_ narrow bandgap are prevented from transferring to the MN layer, as shown in Figure 5b [50], suggesting the potential usage of the light-emitting device.

**Figure 6:**
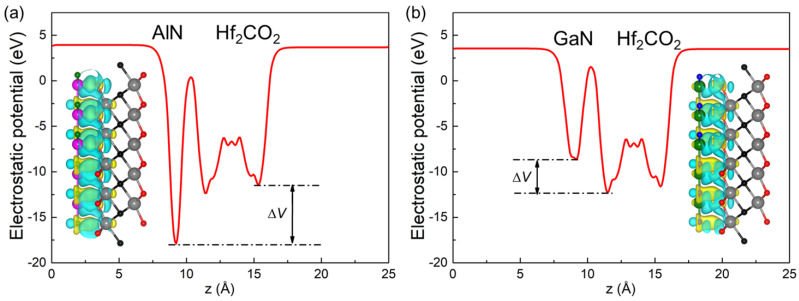
The potential drop for the (**a**) Hf_2_CO_2_/AlN and (**b**) Hf_2_CO_2_/GaN vdW heterostructures between the interface. The yellow demonstration shows the gaining of the electrons, while the cyan one means the loss of electrons. 0.0001 |*e*| is used for the isosurface level.

The interesting properties of the interface for the MXene/MN heterostructure were induced by vdW forces, such as the charge difference density (Δ*ρ*), which is evaluated by:

(2) $$\Delta \rho =\rho_{\mathrm{MXene}/\mathrm{MN}}-\rho_{\mathrm{MXene}}-\rho_{\mathrm{MN}}$$

where *ρ*_MXene/MN_, *ρ*_MXene_ and *ρ*_MN_ show the total charge density of the Hf_2_CO_2_/AlN (or Hf_2_CO_2_/GaN) vdW heterostructure, monolayered Hf_2_CO_2_ and AlN (or GaN), respectively. Figure 6a,b show the difference in charge density between the interface in Hf_2_CO_2_/AlN and Hf_2_CO_2_/GaN vdW heterostructures. It is obvious that AlN (or GaN) acts as an electron donor in contact with Hf_2_CO_2_. In addition, the charge density is redistributed in Hf_2_CO_2_/AlN and Hf_2_CO_2_/GaN vdW heterostructures, which contributes to the formation of electron-rich and hole-rich regions. It is found that there is charge transfer between the two monolayers. The electron transfer of 0.1513 (or 0.0414) |*e*| in the Hf_2_CO_2_/AlN (or Hf_2_CO_2_/GaN) vdW heterostructure is calculated by the Bader-charge analysis method [103]. In addition, we observed that when Hf_2_CO_2_ and MN come into contact and reach the equilibrium position, the potential across the interface of the Hf_2_CO_2_/MN vdW heterostructure decreases to varying degrees due to the charge transfer. The potential of Hf_2_CO_2_/AlN and Hf_2_CO_2_/GaN vdW heterostructures decreases by 6.445 eV and 3.752 eV, respectively, which can also be used as an effective driving force to promote carriers in Figure 5. The charge density differences of the MXene/AlN and MXene/GaN vdW heterostructures compared to the other five stacking configurations are obtained by Figures S3 and S4, respectively, in the Supplementary Materials. It is worth noting that the AlN (or GaN) layer still acts as an electron donor for the Hf_2_CO_2_ layer in the other five stacking configuration heterostructures, and the transferred electrons are calculated in Table S1 in the Supplementary Materials.

**Figure 7:**
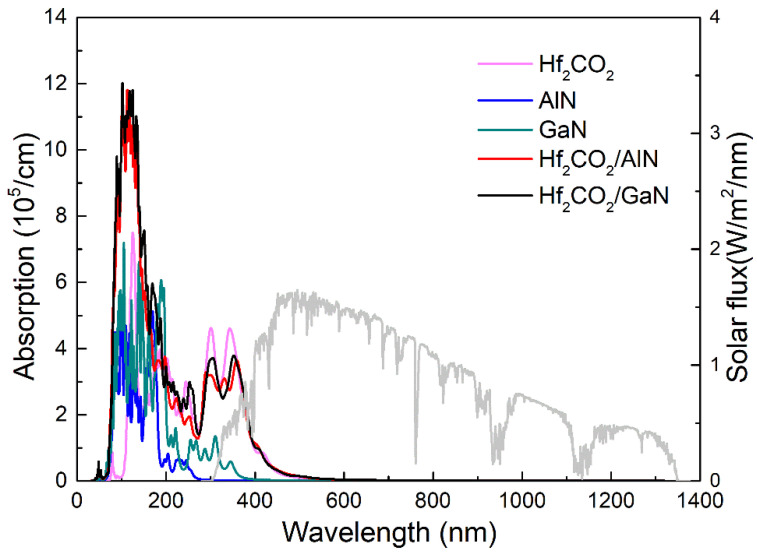
The HSE06 functional obtained optical absorption spectrum for the Hf_2_CO_2_, AlN, GaN monolayers and Hf_2_CO_2_/AlN, Hf_2_CO_2_/GaN vdW heterostructures.

The optical absorption capacity of the Hf_2_CO_2_/MN vdW heterostructure was also investigated. The light absorption capacity of the MXene, MN and the MXene/MN vdW heterostructure is obtained in Figure 7 by the optical absorption spectrum, which is calculated as:

(3) $$\alpha \left(\omega \right)=\frac{\sqrt{2}\omega }{c}{\left\{{\left[\varepsilon_{1}^{2}\left(\omega \right)+\varepsilon_{2}^{2}\left(\omega \right)\right]}^{\frac{1}{2}}-\varepsilon_{1}\left(\omega \right)\right\}}^{\frac{1}{2}}$$

where *ω* is the angular frequency, *α* shows the absorption coefficient and *c* is the speed of light. In addition, *ε*_1_(*ω*) is used to explain the dielectric constant for real parts, and the imaginary one is demonstrated by *ε*_1_(*ω*). It is obvious that the MXene/MN vdW heterostructure possesses the ability to absorb sunlight over a wide range in the visible and NIR regions, which considerably overlaps with the wavelength range of the solar spectrum. Importantly, one can see that the optical performance of the heterostructures is much better than that of AlN and GaN monolayers. Near the wavelength range of visible light, the calculated optical absorption peaks of the Hf_2_CO_2_/AlN and Hf_2_CO_2_/GaN vdW heterostructures are 3.627 × 10^5^ cm^−1^ and 3.778 ×10^5^ cm^−1^, respectively. Furthermore, the Hf_2_CO_2_/AlN and Hf_2_CO_2_/GaN vdW heterostructures also possess another peak value obtained by 1.113 × 10^5^ cm^−1^ and 0.962 × 10^5^ cm^−1^, located at 405 nm and 410 nm, which is higher than that of 0.853 × 10^5^ cm^−1^ for the Hf_2_CO_2_ monolayer. All these results reveal that both Hf_2_CO_2_/AlN and Hf_2_CO_2_/GaN vdW heterostructures have novel optical characteristics. It is worth noting that the calculated optical spectra of these 2D materials in this work do not consider the electron–hole interaction. At present, the *GW*+BSE method has been regarded to be a very crediable method for including the electron–hole interaction, which has been applied in other low-dimensional materials [104,105].

## 4. Conclusions

The structural and electronic properties of Hf_2_CO_2_, AlN and GaN monolayers and their heterostructures are investigated by the DFT method. Both Hf_2_CO_2_/AlN and Hf_2_CO_2_/GaN vdW heterostructures have strong thermal stability and maintain the original structure at 300 K. Importantly, it is found that both Hf_2_CO_2_/AlN and Hf_2_CO_2_/GaN heterostructures are formed by vdW interactions, showing a type-I band structure with a bandgap of 2.006 eV and 1.899 eV, respectively, and they are ideal candidates for light-emitting devices. In addition, the potential of Hf_2_CO_2_/AlN and Hf_2_CO_2_/GaN vdW heterostructures is reduced by 6.445 eV and 3.752 eV, respectively. Furthermore, Hf_2_CO_2_/AlN and Hf_2_CO_2_/GaN vdW heterostructures have excellent light absorption ability, which can provide theoretical support and technical guidance for future light-emitting device materials. For the efficiency of the light-emitting device, some properties play an important role, such as carrier mobility, lifetime, diffusion and light emission capability, which can be studied further.

## Figures and Tables

**Table 1:** The optimized lattice parameter (*a*, Å), bond length (B, Å), binding energy (*E*_binding_, meV/Å^−2^), interface height (*H*, Å) and bandgap (*E*_g_, eV) obtained by HSE06 method for the Hf_2_CO_2_, AlN, GaN monolayers and Hf_2_CO_2_/AlN, Hf_2_CO_2_/GaN heterostructures.

	*a*	B_Hf–C_	B_Hf–O_	B_M–N_	*E*_binding_	*H*	*E*_g_
Hf_2_CO_2_	3.363	2.369	2.132				1.820
AlN	3.127			1.805			4.042
GaN	3.283			1.895			3.203
Hf_2_CO_2_/AlN	3.328	2.354	2.121	1.922	−56.98	1.924	1.826
Hf_2_CO_2_/GaN	3.329	2.355	2.101	1.922	−52.44	2.235	1.734

## Data Availability

The data presented in this study are available upon request from the corresponding author.

## Supplementary Materials

The following are available online at https://www.mdpi.com/article/10.3390/nano11092236/s1, Table S1: The tested results of the energy for the Hf_2_CO_2_/AlN and Hf_2_CO_2_/GaN systems; Figure S1 and Figure S2: The band structure of the MXene/MX heterostructure for other stacking styles; Figure S3 and Figure S4: The charge difference of the MXene/MX heterostructure for other stacking styles; Table S2: The electron transfer between the interface of the Hf_2_CO_2_/AlN and Hf_2_CO_2_/GaN vdW heterostructures.
