# Supplementary material for: Electronic and Optical Properties of Atomic-Scale Heterostructure Based on MXene and MN (M = Al, Ga): A DFT Investigation
Source: Nanomaterials (Basel). 2021 Aug 30;11(9):2236. doi: 10.3390/nano11092236 (PMC8467826; doi:10.3390/nano11092236)
Supplement: Supplementary file 1 [file nanomaterials-11-02236-s001.zip › nanomaterials-1310477-supplementary.pdf]

Electronic and optical properties of atomically scale heterostructure based on MXene and MN (M = Al, Ga): a DFT investigation

Kai Ren[1,*], Ruxin Zheng[1], Peng Xu[1], Dong Cheng[1], Wenyi Huo[1], Jin Yu[2] Zhuoran Zhang[3] and Qingyun Sun[1,*]

[1]School of Mechanical and Electronic Engineering, Nanjing Forestry University, Nanjing, Jiangsu, China

[2]School of Materials Science and Engineering, Southeast University, Nanjing, Jiangsu, China

[3]Center for More-Electric-Aircraft Power System, Nanjing University of Aeronautics and Astronautics, Nanjing 211106, China

Corresponding author: kairen@njfu.edu.cn (Kai Ren), sunqingyun@njfu.edu.cn (Qingyun Sun)

Table S1. The tested results of the energy for the Hf_2CO_2/AlN and Hf_2CO_2/GaN systems.

k-points	Energy (eV)	
	Hf_2CO_2/AlN	Hf_2CO_2/GaN
3	−65.455887	−62.773514
4	−65.601831	−62.979732
5	−65.649084	−63.044835
6	−65.664875	−63.066097
7	−65.670985	−63.074063
8	−65.673149	−63.076884
9	−65.673926	−63.077911
10	−65.674286	−63.078323
11	−65.674416	−63.078500
12	−65.674466	−63.078574
13	−65.674520	−63.078633
14	−65.674534	−63.078639
15	−65.674541	−63.078655
16	−65.674545	−63.078670
17	−65.674557	−63.078658

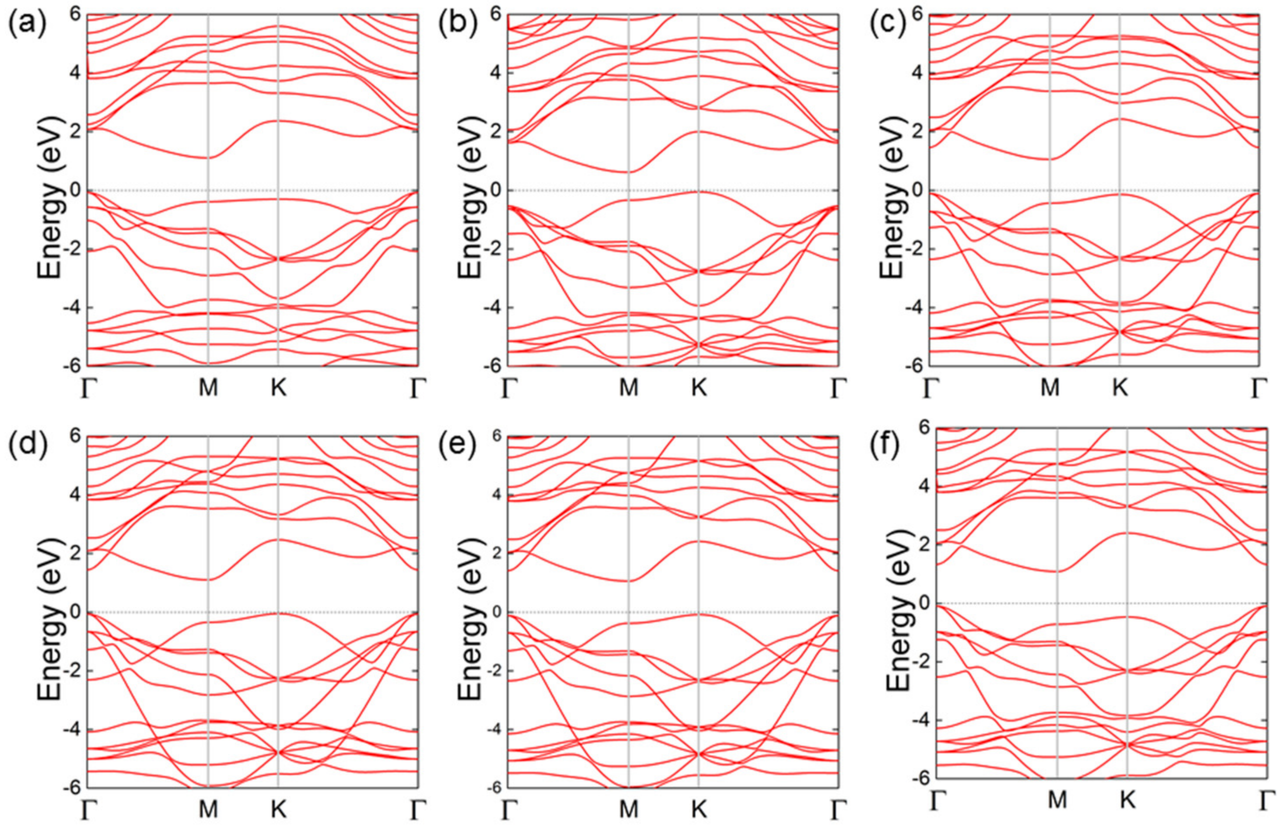

Figure S1. The PBE calculated band structure of the (a) AA, (b) AB, (c) AC, (d) AD, (e) AE and (d) AF stacking configurations of the Hf_2CO_2/AlN vdW heterostructure.

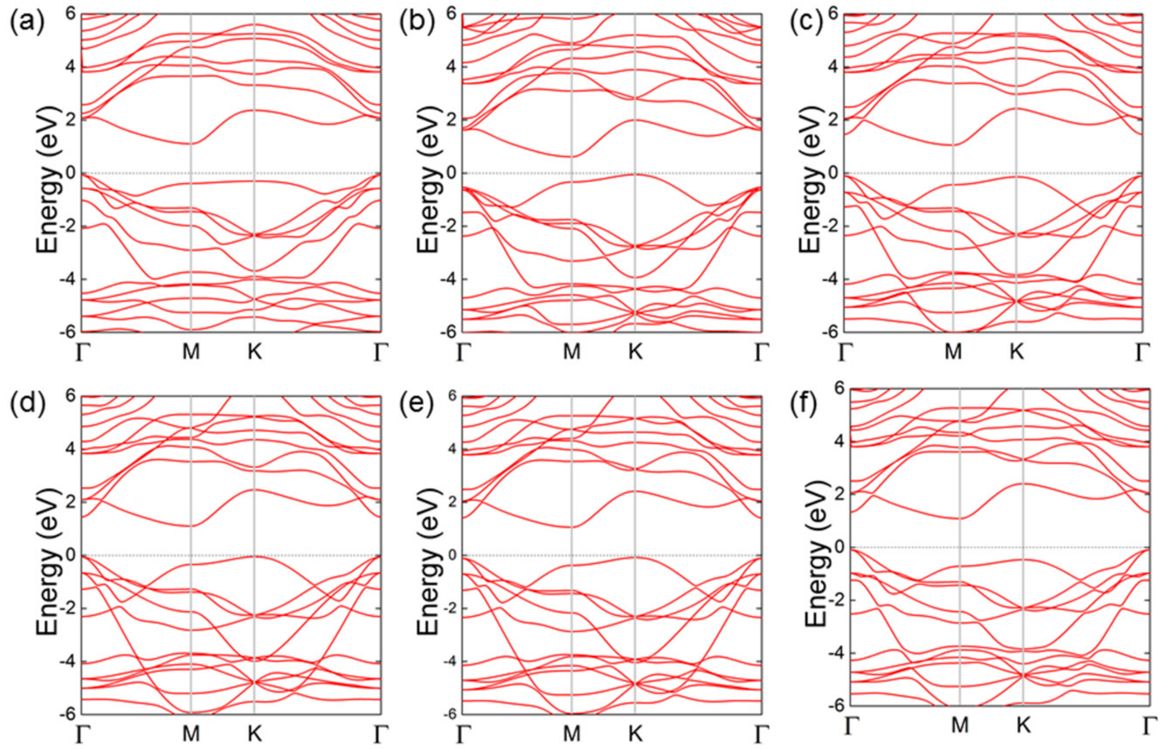

Figure S2. The PBE calculated band structure of the (a) AA, (b) AB, (c) AC, (d) AD, (e) AE and (d)

AF stacking configurations of the Hf_2CO_2/GaN vdW heterostructure.

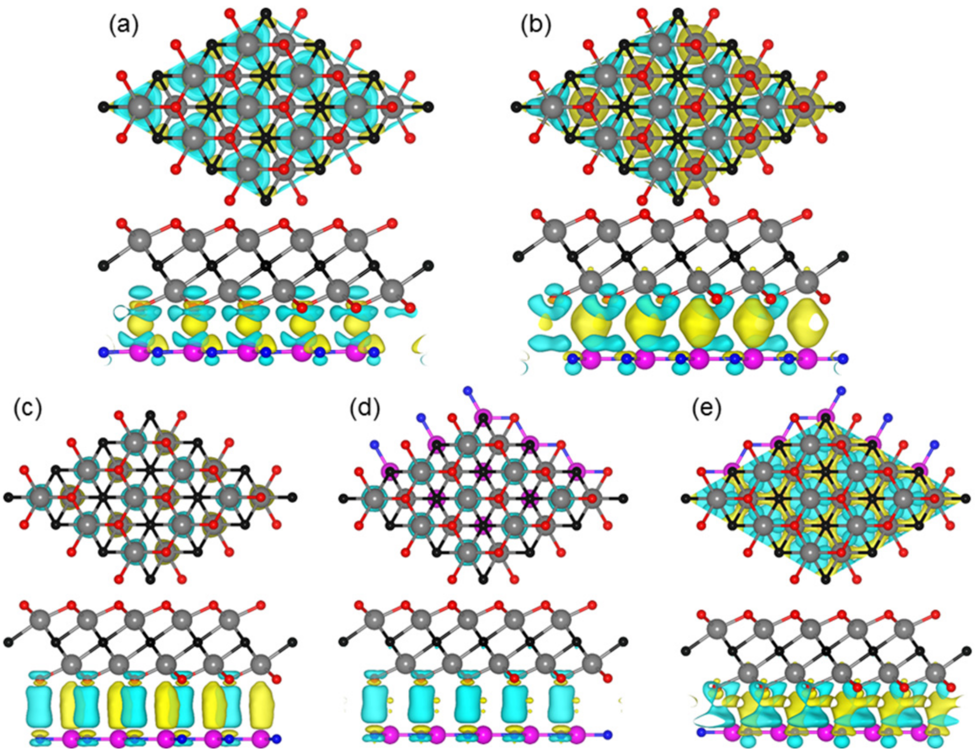

Figure S3. The charge density difference of the (a) AA, (b) AB, (c) AC, (d) AD, (e) AE and (d) AF stacking configurations of the Hf_2CO_2/AlN vdW heterostructure; the yellow demonstration shows the gaining of the electrons while the cyan one means the losing, 0.0001 |*e*| is used for the isosurface level.

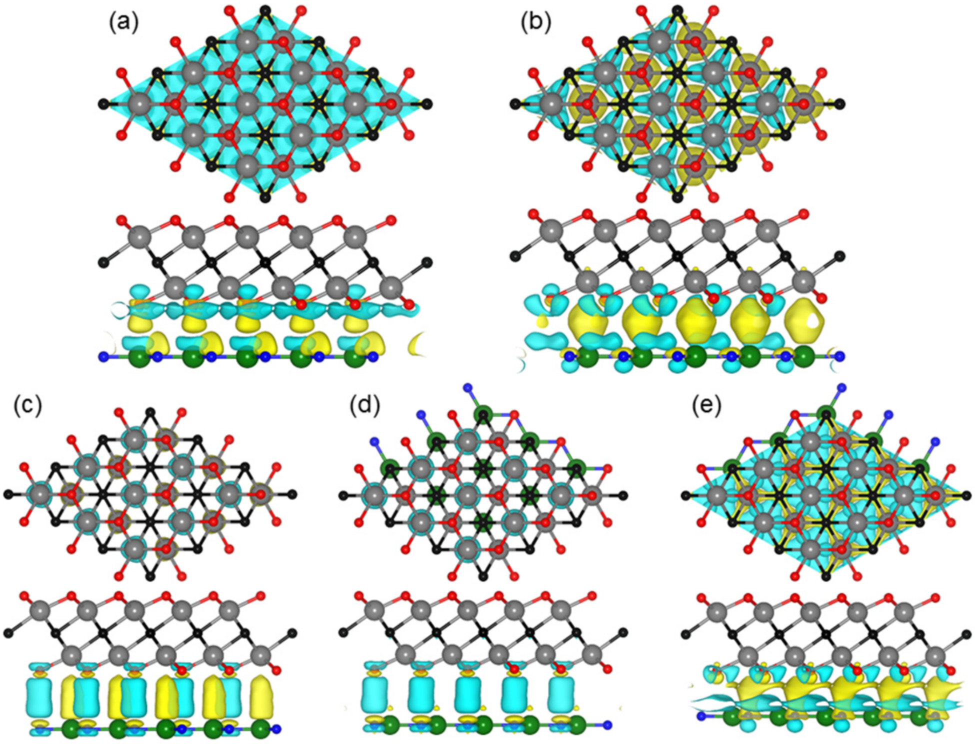

Figure S4. The charge density difference of the (a) AA, (b) AB, (c) AC, (d) AD, (e) AE and (d) AF stacking configurations of the Hf_2CO_2/GaN vdW heterostructure; the yellow demonstration shows the gaining of the electrons while the cyan one means the losing, 0.0001 |*e*| is used for the isosurface level.

Table S2. The electron transfer (unit: |*e*|) between the interface of the Hf_2CO_2/AlN and Hf_2CO_2/GaN vdW heterostructures.

	AA	AB	AC	AD	AE	AF
Hf_2CO_2/AlN	0.1513	0.0928	0.0141	0.0082	0.011	0.0296
Hf_2CO_2/GaN	0.0414	0.0121	0.0114	0.0076	0.0097	0.0205
